# Systematic review of monotherapy with biologicals for children and adults with IgE‐mediated food allergy

**DOI:** 10.1002/clt2.12123

**Published:** 2022-09-27

**Authors:** Debra de Silva, Chris Singh, Stefania Arasi, Antonella Muraro, Torsten Zuberbier, Motohiro Ebisawa, Montserrat Alvaro Lozano, Graham Roberts

**Affiliations:** ^1^ The Evidence Centre London UK; ^2^ The Evidence Centre Wellington New Zealand; ^3^ Bambino Gesù Children's Research Hospital Roma Italy; ^4^ Food Allergy Centre Padua University Hospital Padova Italy; ^5^ Department of Dermatology Charité ‐ Universitätsmedizin Berlin Berlin Germany; ^6^ Sagamihari National Hospital Sagamihara Japan; ^7^ Hospital Sant Joan de Déu Barcelona Spain; ^8^ Paediatric Allergy and Respiratory Medicine University of Southampton Southampton UK

**Keywords:** biologic, etokimab, food allergy, IgE‐mediated, omalizumab

## Abstract

**Background:**

Biological therapies relieve symptoms in allergic inflammatory diseases so we systematically reviewed the evidence about whether biological monotherapy could benefit people with IgE‐mediated food allergy.

**Methods:**

We searched six bibliographic databases from 1946 to 30 September 2021 for randomised and non‐randomised controlled trials about biological monotherapy in people with IgE‐mediated food allergy confirmed by oral food challenge. We used the Grading of Recommendations, Assessment, Development and Evaluation approach to narratively summarise findings from three trials with 118 participants. The studies were too heterogeneous and sparse to conduct meta‐analysis.

**Results:**

We included one randomised trial about etokimab, one about omalizumab and one about the discontinued TNX‐901. All were in people with peanut allergy in the USA, mostly aged 13+ years. There was a trend towards improved tolerance of peanut during treatment, with few side effects. However, we have very low certainty about the evidence due to the small number of trials and participants. No included trial reported on quality of life or cost‐effectiveness.

**Conclusions:**

There is not yet enough certainty to support offering etokimab or omalizumab widely for food allergy. Clinicians may consider the merits for individuals, but large randomised trials with standardised measures are needed to confirm the safety, efficacy and most suitable candidates, doses and durations of treatment before more universal use.

## 1 INTRODUCTION

### 1.1 Background

Immunoglobulin E (IgE)‐mediated food allergy can have a significant impact on people's quality of life, social interactions and nutrition, with a risk of fatal allergic reactions.^1^ Few treatments are available. People are often advised to avoid the allergen, take medication to reduce symptoms and carry adrenaline in case of an anaphylactic reaction. However, elimination diets can be difficult to maintain and there is a risk of severe reactions from accidental exposure.

Researchers are searching for more proactive treatments due to the burden of the condition on patients, their caregivers and healthcare systems. There are promising results from allergen‐specific immunotherapy and biological therapies.^2^, ^3^

Biological therapies are medications derived or synthesised from biological sources. Recombinant monoclonal antibodies are a class of biologicals with targeted application against molecules of intercellular communication, which may be useful in treating inflammatory and allergic diseases.

Biological therapies are effective for people with some inflammatory or allergic responses, such as asthma, dermatitis and chronic rhinosinusitis.^4^, ^5^, ^6^ It has been hypothesised that the same mechanism of action may interfere with IgE synthesis or with the biological mechanisms that establish food allergies.

In contrast to allergen‐specific immunotherapy, which is specific to the culprit food(s) and uses the food allergen in treatment, biologicals are antigen‐independent approaches. They may allow the simultaneous treatment of allergies to multiple foods and other allergenic sources such as inhalants and hymenoptera venom. However, the safety and efficacy of biologicals in IgE‐mediated food allergy remains uncertain.

Claims of effectiveness have been made based largely on observational studies, descriptive reviews or small clinical trials.^7^, ^8^, ^9^, ^10^ These therapies can be costly and can be burdensome for patients, so we wanted to compile the most robust research about biologicals to inform Global Allergy and Asthma European Network (GA^2^LEN) guidelines.^11^ A recent systematic review explored the potential mechanisms of action of biologicals in food allergy,^12^ but we did not identify any systematic reviews of randomised trials of safety or effectiveness.

### 1.2 Objectives

Our systematic review sought to address the following question: what is the efficacy, safety and cost‐effectiveness of biological therapies for children and adults with IgE‐mediated food allergy compared to no active treatment?

We prioritised this question after canvassing people with food allergy, healthcare professionals, teachers and policy makers. No industry representatives were involved in the prioritisation.

Biologicals can be used alone or with allergen immunotherapy. In this review we focused on biologicals used alone. We conducted another review about immunotherapy with or without biologicals, which is published elsewhere.^13^

## 2 METHODS

This review was undertaken by a task force of allergy specialists, primary care doctors, psychologists, other clinicians, patient representatives, teachers and methodologists from 19 countries. The full methods are available in the review protocol, registered with the International Prospective Register of Systematic Reviews (PROSPERO registration: CRD42021250966).

### 2.1 Eligibility criteria

Studies were eligible for the review if they included:

- Population: people with IgE‐mediated food allergy confirmed with oral food challenge.
- Intervention: monotherapy with a biological therapy.
- Comparator: placebo, no intervention or routine management, as long as routine management did not include an active treatment.
- Outcomes: tolerance of food allergen(s) during or after therapy, quality of life, adverse events, severe adverse events and cost‐effectiveness, as defined by the original studies.
- Study types: full publications of randomised controlled trials (hereafter trials), controlled clinical trials or quasi‐randomised trials with a simultaneous comparison group published from the beginning of databases (1946) to 30 September 2021.

We used these inclusion criteria because biologicals can have a high patient burden and be costly. We therefore wanted to summarise the best quality available evidence to inform decision‐making rather than relying on observational studies which may be at higher risk of bias.

### 2.2 Study selection and data extraction

An information specialist (Chris Singh) searched six bibliographic databases using a search strategy developed with clinicians and patient representatives (supporting information S1). The databases were CINAHL, Cochrane Library, EMBASE, ISI Web of Science, MEDLINE and Scopus.

We (Chris Singh, Debra de Silva) searched for other studies by reviewing the reference lists of previous reviews, guidelines and identified studies and by contacting experts in the field. Two methodologists independently screened the titles, abstracts and full text of any studies that were potentially relevant (Chris Singh, Debra de Silva). Shortlisted studies were rescreened by all clinicians, allied health professionals and patient representatives on the task force (all authors and contributors). There was 100% inter‐rater agreement about the studies included.

Two methodologists independently extracted data about study characteristics and outcomes into a bespoke template (Chris Singh, Debra de Silva). Pairs of task force members also extracted and checked data independently (Stefania Arasi, Motohiro Ebisawa, Torsten Zuberbier). We used this process to ensure that data were reviewed by both clinicians and methodologists. A separate arbitrator was available to consider areas of disagreement in the data extraction (Antonella Muraro), but there were no disagreements.

### 2.3 Risk of bias in individual studies

Three clinicians and methodologists independently assessed the risk of bias in individual studies (Stefania Arasi, Chris Singh, Debra de Silva) and this was checked by additional clinicians (Motohiro Ebisawa, Torsten Zuberbier) using the Cochrane Risk of Bias tool 2.^14^ Arbitration was available if needed from a senior clinician (Graham Roberts) but there was agreement in the risk of bias assessments.

### 2.4 Synthesis of results

We used the Grading of Recommendations, Assessment, Development and Evaluation (GRADE) approach to assess the certainty of evidence.^15^ We created evidence profiles to summarise the findings about each outcome (Debra de Silva). We summarised the results narratively because we did not meet the minimum criteria for meta‐analysis set out in our review protocol (PROSPERO registration: CRD42021250966). There were too few studies about each outcome, treatment and population, and the studies were too heterogeneous to pool. We identified only one study about each treatment so could not combine them.

All taskforce members decided on the conclusions by consensus. We used standardised GRADE statements to indicate the effect size and the certainty of the evidence.^16^ Where the certainty of evidence was very low, we used the following terminology, regardless of the effect size: “It is unclear whether [intervention] affects [outcome] because the evidence is very uncertain.”

## 3 RESULTS

### 3.1 Study characteristics

**FIGURE 1:**
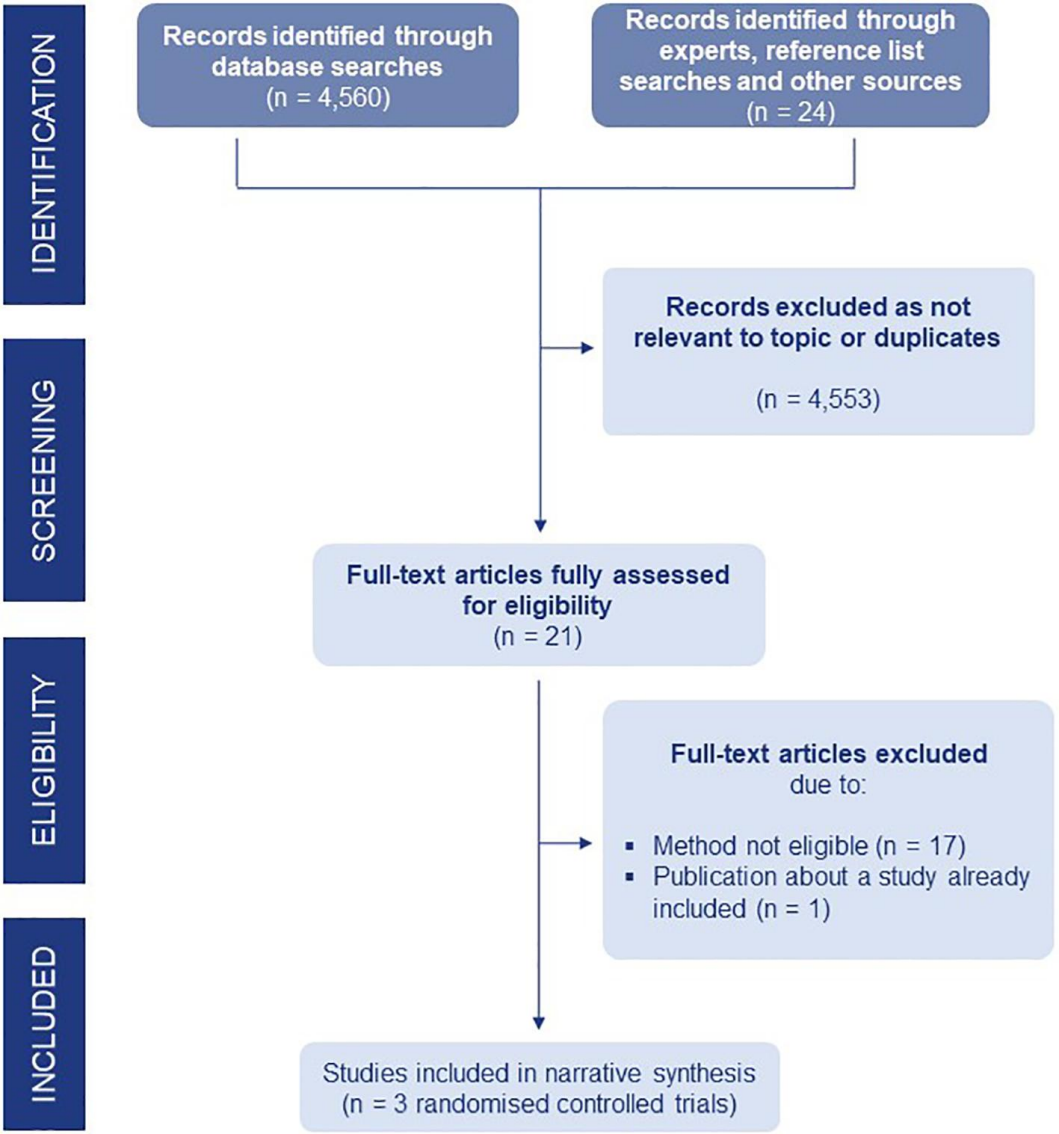
PRISMA diagram showing study selection

We included three randomised controlled trials with 118 participants (Figure 1). One focused on etokimab (an anti‐IL33),^17^ one on omalizumab (anti‐IgE humanised monoclonal antibody developed by recombinant DNA techniques)^18^ and one on TNX‐901 (anti‐IgE humanised IgG1 monoclonal antibody, which was in development but has been discontinued).^19^

All of the trials were conducted in the USA, and all reported industry sponsorship. All focused on people with peanut allergy, mostly moderate to severe. Two trials focused on people aged 13+ years. In the other trial half of participants were aged 5–12 years and the other half were aged 13+ years.

One of the studies was at low risk of bias and two at moderate risk of bias (Table S2). In general, the GRADE certainty of evidence was very low (Table S3). The certainty of evidence was downgraded mainly due to risk of bias, indirectness and imprecision because of small sample sizes and small numbers of studies.

### 3.2 Tolerance of food allergen(s)

Table 1 lists the key findings. Supporting information S3 provides more detail.

It is unclear whether etokimab, omalizumab or TNX‐901 have any impact on how well people tolerate peanut because the evidence is very uncertain. Although there were positive trends in individual studies, there was not enough evidence to draw firm conclusions about the impact of any biological monotherapy on people's ability to tolerate peanut during or shortly after treatment.

One trial found that a single dose of intravenous etokimab increased the proportion able to tolerate a low dose of peanut (275 mg).^17^ One trial of subcutaneous omalizumab was stopped early, with only a small number of participants, due to adverse events during oral food challenge prior to beginning therapy. This study found trends towards improved tolerance which did not reach statistical significance (*p* = 0.054).^18^ One trial of the discontinued TNX‐901 found a significant difference in threshold dose compared to placebo when participants received 450 mg, but not 150 mg or 300 mg subcutaneous doses.^19^

### 3.3 Side effects

It is unclear whether etokimab, omalizumab or TNX‐901 have any impact on adverse events because the evidence is very uncertain (Table 1). The three trials suggested that etokimab, omalizumab and TNX‐901 were generally well tolerated, with no more side effects than placebo.^17^, ^18^, ^19^ Any side effects tended to be mild. However we are not certain about these findings as two out of the three studies were at moderate risk of bias, the sample sizes were small and the inclusion criteria and measurement strategies varied.

**TABLE 1:** Summary of findings about biologicals for IgE‐mediated food allergy

Intervention	Regimen	Population	Tolerance	Safety	Certainty of evidence	Studies and participants
Etokimab (anti‐IL33)	Single dose of etokimab, 300 mg/100 ml i.v.	13+ years with peanut allergy in USA	Significant increase in ability to tolerate 275 mg peanut protein at day 15 (73% intervention vs. 0% placebo, *p* < 0.01).	No significant difference in adverse effects (80% intervention vs. 100% placebo, *p* > 0.05). The most frequent reported treatment emergent effect was headache. No severe reactions.	Very low	1 randomised trial, *n* = 20^15^
Omalizumab (anti‐IgE humanised monoclonal antibody developed by recombinant DNA techniques).	Dose determined according to asthma indication based on total IgE levels and body weight. subcutaneous treatment was for 20–22 weeks every 2–4 weeks.	5–12 and 13+ years with peanut allergy in USA	No significant difference in proportion that could tolerate >1000 mg peanut at 24 weeks (44% intervention vs. 20% placebo, *p* > 0.05) or change from baseline threshold (80.9 times intervention vs. 4.07 times placebo, *p* = 0.054)	No significant difference in adverse events (77% intervention vs. 89% placebo, *p* > 0.05). no severe reactions.	Very low	1 randomised trial, *n* = 14^16^
TNX‐901 (anti‐IgE humanised IgG1 monoclonal antibody, which was in development but has been discontinued)	150, 300, or 450 mg subcutaneously every 4 weeks for 4 doses.	13+ years with peanut allergy in USA	Significant difference in mean increase in threshold dose between 450 mg dose and placebo (2627 vs. 710 mg, *p* < 0.01). No significant differences for other doses.	No significant difference between groups in overall adverse events or severe adverse events (*p* > 0.05).	Very low	1 randomised trial, *n* = 84^17^

*Note*: All studies compared with placebo. ‘Significant’ is a statistically significant difference at the 95% level of confidence.

### 3.4 Other outcomes

None of the included studies reported on quality of life or cost‐effectiveness.

## 4 DISCUSSION

### 4.1 Summary of evidence

Around 6% of people suffer from IgE‐mediated food allergy^20^ and the prevalence is increasing in many regions.^21^ Therefore researchers are searching for proactive treatments to reduce the burden for individuals, families and communities. Biological monotherapy could be useful because immunotherapy protocols are cumbersome, take time to see a clinical benefit and are allergen‐specific.^2^ However, we found little robust evidence about the efficacy and safety of biological monotherapy for IgE‐mediated food allergy. Whilst there are many observational studies and opinion pieces describing potential benefits,^9^ there is not yet sufficient robust, randomised controlled evidence to substantiate these claims. The certainty of evidence is very low, though there were positive trends and biologicals appeared to be well‐tolerated.

All of the studies we identified focused on people with peanut allergy, largely aged 13+ years, so potential benefits in younger children and those with other types of food allergy remain uncertain.

### 4.2 Comparison with previous research

It is difficult to rely on the plethora of non‐randomised and non‐controlled studies about biologicals due to concerns about generalisability and confounding. Our review differs from previous reviews and observational studies because it focused on the highest quality published evidence. It excluded studies at high risk of bias, such as abstracts, posters and unpublished research, studies that did not use food challenges to confirm food allergy before treatment and those without a simultaneous control group. This means that our review is more conservative in its findings compared to previous studies, but also more robust.

### 4.3 Implications for research

There is much left to learn about the efficacy and safety of biological monotherapy, patient preferences, cost‐effectiveness, the optimal duration and dose and the most suitable candidates. Given the positive findings from observational studies, there is a need for well‐designed controlled studies that use standardised definitions for tolerance and adverse events and focus on a wider range of food allergies and age groups. Further research may also explore whether monotherapy has any benefits over combining biologicals with allergen immunotherapy. Clinicians should actively encourage people with food allergy to take part in trials.

### 4.4 Strengths and limitations

Our review was conducted by a diverse group of clinicians, allied health professionals, patient representatives and researchers from around the world. This means we took into account a wide range of perspectives and weighed up the findings on clinical and methodological grounds.

However, our conclusions are limited by the scarcity of good quality studies meeting our inclusion criteria. It is difficult to draw conclusions about efficacy, safety and cost‐effectiveness based on just three studies of different medications, one of which is now obsolete. We could not meta‐analyse the findings, as there was just one trial on each therapy.

## 5 CONCLUSIONS

Biological monotherapy might be useful for some individuals with food allergy, especially given the potentially positive safety profile, lack of allergen specificity and potential to treat multiple allergic conditions at once. However, the patient burden and cost means good quality research is needed before considering biologicals more widely for people with food allergy. GA^2^LEN's food allergy management guidelines used the findings from this review alongside expert opinion and other evidence to suggest practical implications for health professionals, teachers and families.^11^

## AUTHOR CONTRIBUTIONS

All authors conceptualised the work, provided comments and approved the review for submission. In addition, Chris Singh, Debra de Silva, Stefania Arasi, Motohiro Ebisawa and Torsten Zuberbier extracted data and undertook risk of bias assessments. Debra de Silva constructed narrative summaries and prepared initial drafts. Antonella Muraro, Stefania Arasi and Graham Roberts managed the process.

## CONTRIBUTORS

Susanne Halken, Hans Christian Andersen Children's Hospital, Odense University Hospital, Denmark

Margitta Worm, Charité ‐ Universitätsmedizin Berlin, Germany

Montserrat Fernandez Rivas, Hospital Clínico San Carlos, Spain

Alessandro Fiocchi, Bambino Gesù Children's Hospital IRCCS, Italy

Amena Warner, Allergy UK

Angel Sanchez, AEPNAA Spanish Association for People with Food and Latex Allergy, Spain

Anna Nowak‐Wegrzyn, NYU Langone Health, USA

Antoine Deschildre, University Lille, France

Antonella Cianferoni, University of Florence, Italy

Audrey Dunn‐Galvin, University College Cork, Ireland

Barbara Ballmer, UniversitätsSpital Zürich, Switzerland

Berber Vlieg‐Boerstra, OLVG Hospital, the Netherlands

Bertine Flokstra‐de Blok, University of Groningen, the Netherlands

Bright Nwaru, University of Gothenburg, Sweden

Carina Venter, Children's Hospital Colorado, USA

Carla Jones, Allergy UK

Caroline Nilsson, Karolinska Institutet and Sachs' Children and Youth Hospital, Sweden

Carsten Bindslev‐Jensen, Odense University Hospital, Denmark

Celine Demoulin, Association Française pour la Prévention des Allergies Siège social, France

Clare Mills, University of Manchester, UK

Ekaterina Khaleva, University of Southampton, UK

Gary Wong, Chinese University of Hong Kong, Hong Kong

Giovanni Pajno, Policlinico Hospital‐University of Messina, Italy

Hania Szajewska, Medical University of Warsaw, Poland

Hasan Arshad, University of Southampton, UK

Hugh Sampson, Mount Sinai School of Medicine, USA

Jennifer Gerdts, Food Allergy Canada, Canada

Josefine Gradman, Hans Christian Andersen Children's Hospital, Denmark

Kate Grimshaw, Salford Care Organisation, UK

Kirsten Beyer, Charité‐Universitätsmedizin Berlin, Germany

Lars K. Poulsen, Copenhagen University Hospital, Denmark

Liz Angier, University of Southampton, UK

Lynne Regent, Anaphylaxis Campaign, UK

Marcia Podestà, Food Allergy Italia, Italy

Matthew Geromi, The Evidence Centre, USA

Mika Makela, Helsinki University Hospital, Finland

Nicolette W. de Jong, University Medical Centre Rotterdam, the Netherlands

Pablo Rodríguez del Río, Niño Jesus University Children's Hospital, Spain

Paul Turner, Imperial College, UK

Pete Smith, Griffith University School of Medicine, Australia

Philippe Begin, Univerisite de Montreal, Canada

Richard Loh, Perth Children's Hospital, Australia

Robert Wood, John Hopkins Bloomberg School of Public Health, USA

Ronald Van Ree, Amsterdam UMC, the Netherlands

Rosan Meyer, Imperial College, England

Sabine Schnadt, DAAB, Deutscher Allergie‐und Asthmabund e.V, Germany

Susanne Lau, Charité ‐ Universitätsmedizin Berlin, Germany

Ulugbek Nurmatov, Cardiff University, UK

## CONFLICT OF INTERESTS

Debra de Silva, no conflicts declared. Organisation received GA^2^LEN funding to help prepare review. Chris Singh, no conflicts declared. Organisation received GA^2^LEN funding to help prepare review. Stefania Arasi, no conflicts declared. Antonella Muraro, Personal fees: DVB, Aimmune, Mylan, ALK, Nestle. Grant: Aimmune Torsten Zuberbier, Personal fees: Abbvie, ALK, Almirall, Bayer, Bencard, Berlin Chemie, Faes, HAL, Leti, L’Oreal, Meda, Menarini, Merck, Novartis, Pfizer, Sanofil, Stallergenes, Takeda, UCB. Grant: Henkel. Motohiro Ebisawa, Personal fees: DBV, Mylan. Montserrat Alvaro Lozano, no conflicts declared Graham Roberts. No conflicts declared. Editor: Editor in Chief Clinical & Experimental Allergy.

## Supporting information

Supporting information S1

Table S2

Table S3
