# Supplementary material for: Systematic review of monotherapy with biologicals for children and adults with IgE‐mediated food allergy
Source: Clin Transl Allergy. 2022 Sep 27;12(9):e12123. doi: 10.1002/clt2.12123 (PMC9515515; doi:10.1002/clt2.12123)
Supplement: Supplementary file 1 — Supporting information S1 [file CLT2-12-e12123-s003.docx]

Online supplement S1: Search strategy

**Search strategy for MEDLINE and EMBASE**

1. exp Food Hypersensitivity/

2. exp Milk Hypersensitivity/

3. exp Egg Hypersensitivity/

4. exp Peanut Hypersensitivity/

5. exp Tree nut Hypersensitivity/

6. exp Nut Hypersensitivity/

7. ((food or Oral Allergy Syndrome or milk or egg or peanut or arachis hypogaea or tree nut or hazelnut or brazil nut or walnut or chestnut or pistachio or almond or legumes or wheat or rice or soy or fish or seafood or shellfish or shrimp or lobster or crab or crawfish or kiwi or apple or peach or apricot or cherry or pear or plum or tomato or green pea or potato or carrot or parsley or celery or additives) adj3 (allerg* or hypersensitivit*)).mp.

8. or/1-7

9. Biologic*.mp.

10. (omalizumab or TNX-901 or dupilumab or reslizumab or mepolizumab or benralizumab or ligelizumab or ANB020 or etokimab or tezepelumab or quilizumab or tralokinumab or nemolizumab).mp.

11. Or/9-10

12. exp Intervention Studies/

13. Intervention Studies.mp.

14. Experimental stud*.mp.

15. exp Clinical Trial/

16. Trial.mp.

17. Clinical Trial.mp.

18. Randomi?ed Controlled Trial.mp.

19. exp Placebos/

20. Placebos.mp.

21. exp Random Allocation/

22. Random Allocation.mp.

23. exp Double-Blind Method/

24. Double-Blind Method.mp.

25. Double-Blind design.mp.

26. exp Single-Blind Method/

27. Single-Blind Method.mp.

28. Single-Blind design.mp.

29. Triple-Blind Method.mp.

30. Random*.mp.

31. Quasi random*.mp.

32. Controlled clinical trial.mp.

33.. Cost.mp.

34. Exp Health care Costs/

35. Economic evaluation*.mp.

36. ((cost effective* adj1 analys*) or cost minimi?ation analys* or cost benefit analys* or cost utility analys* or cost consequence analys* or finances).mp.

37. Quality of life.mp.

38. Efficacy.mp.

39. Effective*.mp.

40. Or/12-39

56. 8 and 11 and 40

[mp=title, original title, abstract, name of substance word, subject heading word, unique identifier]

**Search strategy for CINAHL, Cochrane Library, ISI Web of Science and Scopus**

(Food hypersensitivity or food allergy or Oral Allergy Syndrome or milk allergy or egg allergy or nut allergy or peanut allergy or arachis hypogaea allergy or tree nut allergy or hazelnut allergy or legumes allergy or wheat allergy or soy allergy or fish allergy or seafood allergy or shellfish allergy or kiwi allergy or apple allergy or peach allergy or additives hypersensitivity or additives allergy)

AND

(biologic* or omalizumab or TNX-901 or dupilumab or reslizumab or mepolizumab or benralizumab or ligelizumab or ANB020 or etokimab or tezepelumab or quilizumab or tralokinumab or nemolizumab)

AND

(Intervention stud* or experimental stud* or trial or clinical trial* or randomi* controlled trial or random allocation or single blind method or double blind method or triple blind method or random* or quasi* or controlled clinical trial or economic evaluation* or cost effective* analys* or cost analys* or cost benefit analys* or cost utility analys* or cost consequence analys* or finances or quality of life or efficacy or desensiti* or sustained unresponsiveness)
