# Supplementary material for: Systematic review of monotherapy with biologicals for children and adults with IgE‐mediated food allergy
Source: Clin Transl Allergy. 2022 Sep 27;12(9):e12123. doi: 10.1002/clt2.12123 (PMC9515515; doi:10.1002/clt2.12123)
Supplement: Supplementary file 2 — Table S2 [file CLT2-12-e12123-s001.docx]

Online supplement S2: Risk of bias assessment and study characteristics

Summary of risk of bias assessment

| Citation | Randomisation process risk of bias | Risk of bias due to assignment / deviations from intended intervention | Risk of bias due to missing outcome data | Risk of bias due to outcome measurement | Risk of bias due to selection of reported result | Overall risk of bias |
| --- | --- | --- | --- | --- | --- | --- |
| Chinthrajah S, Cao S, Liu C, Lyu SC, Sindher SB, Long A, Sampath V, Petroni D, Londei M, Nadeau KC. Phase 2a randomized, placebo-controlled study of anti-IL-33 in peanut allergy. JCI Insight 2019 ;4(22):e131347. | Unclear  Randomisation process not described apart from 3:1 ratio. Some participant characteristics may be unbalanced e.g. gender (47% female in intervention group vs 60% controls); peanut specific IgE at baseline; median cumulative tolerated dose at baseline. Lack of balance not through fault in randomisation process, but small numbers likely contributed so results may be at high risk of bias due to different characteristics of each group. | Low  All participants received planned treatment. Multicentre study. All standardised oral food challenge results were reviewed by an independent, blinded expert reviewer (though uncertain whether rest of study team were blinded). States all work done in research facilities with independent review, but two food challenges were open food challenges rather than double-blind placebo-controlled food challenges. | Moderate  Some loss to follow up at 45 day follow up. Follow up was convenience sample based on people's ability to visit the clinic in their work schedules for challenge. Convenience sample has risk of bias. | Moderate  Blinded outcome assessment, however two food challenges were open, which could make a difference when sample size is small. Authors do not specify which group(s) had open challenges. Oral food challenge results were reviewed by an independent, blinded expert reviewer. Adverse effect data mostly based on participant self-reports, which can be variable and may not measure events of interest. | Moderate  Analyses conducted based on pre-defined analysis plan. All planned safety analyses appear to be presented. However, P-values not reported for adverse events analysis even though safety was a key focus of the study. | **Moderate** |
| Leung DYM, Sampson HA, Yunginger JW, Burks Jr AW., Schneider LC, Wortel CH, Davis FM, Hyun JD, Shanahan WR. Effect of anti-IgE therapy in patients with peanut allergy. N Engl J Med 2003;348:986-93. | Low  Patients were randomly assigned in groups of 28 and in a 3:1 ratio to receive 150 mg, 300 mg, or 450 mg of TNX-901 or placebo. Central randomisation performed in blocks of four per site. Enrolment at each dose level was completed before enrolment at the next level began. No information provided about generation or concealment methods. | Low   Double-blind study with patients randomly assigned in a 3:1 ratio with three different doses or placebo. A pharmacist reconstituted with 1 ml of sterile water in an unblinded fashion and placed the solution in a syringe, which was masked to prevent study personnel from identifying the contents, for subcutaneous injection. | Low   Little missing data with 81 of 84 patients completing study. What is missing unlikely to have an impact on trial result. | Low   Double-blind study. Efficacy was assessed in all patients with a double-blind, placebo-controlled oral food challenge. | Low   Data from all participating centres were sent to independent group. All data were entered and locked before analysis began. Data analysed by separate team. | **Low** |
| Sampson HA, Leung DY, Burks AW, Lack G, Bahna SL, Jones SM, Wong DA. A phase II, randomized, double‑blind, parallel‑group, placebo‑controlled oral food challenge trial of Xolair (omalizumab) in peanut allergy. J Allergy Clin Immunol 2011 ;127(5):1309-10.e1. | Unclear  Randomisation process not described. Randomized 2:1 (omalizumab: placebo) to double-blind treatment. Generation and allocation concealment unknown. However, process meant thee were 5 in control group and 9 in intervention group, with differences between groups in baseline characteristics: average age (27 vs 16 placebo), gender (f=20% vs 56% placebo) and proportion age 6-12 years (40% vs 56% placebo) | Low  Assignment was double blind. | High  Study intended to randomize 150 people but was stopped early due to recommendation of the Data Safety Monitoring Committee because of the severity of 2 anaphylactic reactions that occurred during oral food challenges before the administration of the study drug. This meant that only 14 people reached the study’s primary endpoint before the trial was discontinued. The trial appears to have been implemented well in those 14 people, but there is still a high risk of bias due to missing outcome data / limited sample size / early closure. | Low   All participants were assessed in the same way. Approach appears appropriate. Not influenced by prior knowledge of allocation. | Low   Analyses conducted based on pre-defined analysis plan. | **Moderate** |

Characteristics of individual studies

| Citation | Country | Funding source | Total participants | Intervention group no. | Age | Allergy type | Severity of allergy | Biological tested | Biological dose and duration | Comparator |
| --- | --- | --- | --- | --- | --- | --- | --- | --- | --- | --- |
| Chinthrajah et al. Phase 2a randomized, placebo-controlled study of anti-IL-33 in peanut allergy. JCI Insight 2019 ;4(22):e131347. | USA | US National Institutes for Health, AnaptysBio, Hartman Vaccine Fund, Stanford University Sean N. Parker Center for Allergy and Asthma Research | 20 | 15 | Adults. Median age (range): intervention 27 years (19 to 54); placebo 22 years (18 to 50) | Peanut | Not specified  Appears moderate. | Etokimab | Single dose of etokimab, 300mg/100 mL i.v. | Placebo |
| Leung et al. Effect of anti-IgE therapy in patients with peanut allergy. N Engl J Med 2003;348:986-93. | USA | Tanox, US Peanut Board, US Peanut Foundation, US Institutes of Health National Center of Research, Mount Sinai School of Medicine, Mayo Foundation. Sponsor did not limit the investigators’ right to publish the results. | 84 | 61 150mg = 19 300mg = 19 450mg = 21 | 13+ years  Eligible: 12 to 60 years, included 13 to 59 | Peanut | Moderate to severe | TNX-901 (humanized IgG1 monoclonal antibody against IgE) | 150mg, 300mg, or 450mg of TNX-901 subcutaneously every 4 weeks for 4 doses. | Placebo |
| Sampson et al. A phase II, randomized, double‑blind, parallel‑group, placebo‑controlled oral food challenge trial of Xolair (omalizumab) in peanut allergy. J Allergy Clin Immunol 2011 ;127(5):1309-10.e1. | USA | Genentech | 14 | 9 | Mixed 5 to 12 (50%) and 13+ years (50%)  Range 6 to 75 years | Peanut | Not specified | Omalizumab | Dose based on total IgE levels and body weight. Treatment was 20 to 22 weeks every 2 to 4 weeks. Dose was a minimum of 0.016 mg/kg/IgE (IU/mL) every 4 weeks. Those requiring more than a 300mg dose had the dose divided and given every 2 weeks. | Placebo |
