# Supplementary material for: Systematic review of monotherapy with biologicals for children and adults with IgE‐mediated food allergy
Source: Clin Transl Allergy. 2022 Sep 27;12(9):e12123. doi: 10.1002/clt2.12123 (PMC9515515; doi:10.1002/clt2.12123)
Supplement: Supplementary file 3 — Table S3 [file CLT2-12-e12123-s002.docx]

Online supplement S3: Summary of findings tables and evidence profiles

1. Combined summary of findings: any type of biological monotherapy

**Summary of findings**

| **Any biological monotherapy** | | | | |
| --- | --- | --- | --- | --- |
| **People:** Children and adults with peanut allergy  **Settings:** Hospital (outpatient), USA  **Intervention:** Single dose intravenous etokimab (300mg/100 mL) OR omalizumab for 20-22 weeks every 2-4 weeks OR 150mg, 300mg, or 450mg of TNX-901 subcutaneously every 4 weeks for 4 doses  **Comparison:** Placebo | | | | |
| **Outcomes** | **Summary** | **Certainty of evidence** | **Number of studies (participants)** | **Findings of individual studies** |
| Ability to consume peanut: % able to tolerate defined dose | There is low certainty of evidence about the effect of monotherapy with biological medicines on the ability to tolerate peanut.  Two out of three RCTs found no change in the proportion able to tolerate a defined dose. One RCT found that a single dose of intravenous etokimab increased the proportion able to tolerate a low dose of peanut (275mg) (Chinthrajah 2019). Two other RCTs found no significant differences after omalizumab (Sampson 2011) or TNX-901 (Leung 2003). | Very low | 3 RCTs  (total n = 118)  Etokimab: 1 RCT (n = 20)  Omalizumab: 1 RCT (n = 14)  TNX-901: 1 RCT (n = 84) | One small RCT found 73% of adults taking a single dose of intravenous etokimab tolerated 275mg peanut protein at 15 days compared to 0% of placebo (p<0.01) (Chinthrajah 2019).  One very small RCT found that omalizumab did not increase the proportion of children and adults able to tolerate 1000mg peanut protein at 24 weeks (44% omalizumab vs 20% placebo, p>0.05) (Sampson 2011).  One RCT found that TNX-901 did not increase the proportion of adults able to tolerate 8000mg peanut protein at 14-16 weeks (0% 150mg TNX-901 vs 21% 300mg TNX-901 vs 24% 450mg TNX-901 vs 4% placebo, p>0.05) (Leung 2003). |
| Change in median or mean cumulative tolerated dose of peanut protein compared to baseline | The impact of biological medicines on the dose of peanut tolerated is unclear because the certainty of evidence is very low.  One RCT found an improvement with etokimab and another with 450mg TNS-901. Another RCT found no significant difference with omalizumab. | Very low | 3 RCTs  (total n = 118)  Etokimab: 1 RCT (n = 20)  Omalizumab: 1 RCT (n = 14)  TNX-901: 1 RCT (n = 84) | One small RCT found that adults receiving a single dose of intravenous etokimab were able to tolerate a higher median dose of peanut protein at 15 days compared to those receiving placebo (increase in median dose compared to baseline: etokimab 100mg vs placebo 50mg, p<0.05) (Chinthrajah 2019).  One very small RCT found no statistically significant improvement from omalizumab in the maximum dose of peanut tolerated by children and adults compared to baseline, but there were positive trends (p>0.05) (Sampson 2011).  One RCT found that a 450mg dose of TNX-901 increased adult’s threshold of sensitivity to peanut from about half a peanut at baseline (178mg) to almost nine peanuts by 18-20 weeks (2805mg). This was statistically significant compared to placebo (p<0.001). Lower doses of TNX-901 did not have a statistically significant effect. (Leung 2003). |

| **Outcomes** | **Summary** | **Certainty of evidence** | **Number of studies (participants)** | **Findings of individual studies** |
| --- | --- | --- | --- | --- |
| Adverse events | The impact of biological medicines on adverse events is unclear because the certainty of evidence is very low.  Three RCTs suggested that biologicals were generally well tolerated. | Very low | 3 RCTs  (total n = 118)  Etokimab: 1 RCT (n = 20)  Omalizumab: 1 RCT (n = 14)  TNX-901: 1 RCT (n = 84) | One small RCT found that 20% of adults receiving a single dose of intravenous etokimab had mild adverse events compared with 40% of placebo. 60% of the etokimab group had moderate events versus 100% of placebo (p not reported) (Chinthrajah 2019).  One very small RCT found no difference in mild to moderate adverse events reported in children and adults taking omalizumab versus placebo (77% vs 89% placebo, p>0.05) (Sampson 2011).  One RCT found no difference in the incidence and type of adverse events between 150mg, 300mg or 450mg subcutaneous TNX-901 versus placebo (Leung 2003). |
| Severe or systemic adverse events | The impact of biological medicines on severe or systemic adverse events is unclear because the certainty of evidence is very low.  Three RCTs found that biologicals were not associated with significantly more severe adverse events than placebo. | Very low | 3 RCTs  (total n = 118)  Etokimab: 1 RCT (n = 20)  Omalizumab: 1 RCT (n = 14)  TNX-901: 1 RCT (n = 84) | One small RCT found no severe or systemic adverse events in adults taking a single dose of etokimab or placebo (p>0.05) (Chinthrajah 2019).  One very small RCT found no systemic adverse events in children and adults taking omalizumab or placebo (Sampson 2011).  One RCT found no difference in the incidence and type of systemic adverse events was similar between 150mg, 300mg or 450mg subcutaneous TNX-901 vs placebo  (Leung 2003). |

**Evidence profile**

| Studies (participants) | Risk of bias | Inconsistency | Indirectness | Imprecision | Publication bias | Overall certainty |
| --- | --- | --- | --- | --- | --- | --- |
| **Ability to consume peanut: % able to tolerate defined dose** | | | | | | |
| 3 RCTs  (total n = 118)  Etokimab: 1 RCT (n = 20)  Omalizumab: 1 RCT (n = 14)  TNX-901: 1 RCT (n = 84) | Reduced grade by one level as two out of three studies are at moderate risk of bias | No grade change. Two out of three studies found consistent result, but number of studies too small to draw conclusions about consistency. | Reduced grade by one level. Studies all from one country. Studies each focused on different therapy. Varied inclusion criteria. | Reduced grade by one level due to small sample sizes, lack of reporting of p-values in some cases and wide confidence intervals. | No grade change, but publication bias possible given small number of studies and all industry sponsored. | **Very low**  One study found large effect and dose response relationship but not consistent enough to grade up one level. |
| **Ability to tolerate peanut: change in mean or median cumulative tolerated dose** | | | | | | |
| 3 RCTs  (total n = 118)  Etokimab: 1 RCT (n = 20)  Omalizumab: 1 RCT (n = 14)  TNX-901: 1 RCT (n = 84) | Reduced grade by one level as two out of three studies are at moderate risk of bias | No grade change. Two out of three studies found consistent result, but number of studies too small to draw conclusions about consistency. | Reduced grade by one level. Studies all from one country. Studies each focused on different therapy. Varied inclusion criteria. | Reduced grade by one level due to small sample sizes, lack of reporting of p-values in some cases and wide confidence intervals. | No grade change, but publication bias possible given small number of studies and all industry sponsored. | **Very low**  No reason to grade up based on effect size, dose response or potential effect of confounders |
| **Adverse events and systemic/severe adverse events** | | | | | | |
| 3 RCTs  (total n = 118)  Etokimab: 1 RCT (n = 20)  Omalizumab: 1 RCT (n = 14)  TNX-901: 1 RCT (n = 84) | Reduced grade by one level as two out of three studies are at moderate risk of bias | No grade change. Consistent findings across three studies. | Reduced grade by one level. Studies all from one country. Studies each focused on different therapy. Varied inclusion criteria. | Reduced grade by one level due to small sample sizes, lack of reporting of p-values in some cases and wide confidence intervals. | No grade change, but publication bias possible given small number of studies and all industry sponsored. | **Very low**  No reason to grade up based on effect size, dose response or potential effect of confounders |

Note: For an explanation of the GRADE approach to assessing the certainty of evidence, see: Schünemann HJ, Higgins JPT, Vist GE, Glasziou P, Akl EA, Skoetz N, Guyatt GH. Chapter 14: Completing ‘Summary of findings’ tables and grading the certainty of the evidence. In: Higgins JPT, Thomas J, Chandler J, Cumpston M, Li T, Page MJ, Welch VA (editors). Cochrane Handbook for Systematic Reviews of Interventions version 6.2 (updated February 2021). Cochrane, 2021. Available from [www.training.cochrane.org/handbook](http://www.training.cochrane.org/handbook). This note applies to all Evidence Profiles in this supplementary material.

1. Monotherapy with etokimab

**Summary of findings**

| **Etokimab single dose** | | | | |
| --- | --- | --- | --- | --- |
| **People:** Adults with mild to moderate peanut allergy  **Settings:** Hospital, USA  **Intervention:** Single dose of etokimab, 300mg/100 mL intravenous  **Comparison:** Placebo | | | | |
| **Outcomes** | **Summary** | **Certainty of evidence** | **Number of studies (participants)** | **Findings of individual studies** |
| Ability to consume peanut: % with cumulative tolerated dose of 275mg peanut protein on day 15 | Single dose intravenous etokimab may increase ability to consume peanut at 15 days. One small RCT found an absolute change in the ability to tolerate 275mg peanut protein after 15 days was 73% etokimab vs 0% placebo (p<0.01). | Low | 1 RCT (n = 20) | One RCT found that a higher proportion of adults receiving a single dose of intravenous etokimab (300mg/100 mL) were able to tolerate 275mg peanut protein at 15 day compared to those receiving placebo: 73% (11/15) etokimab vs 0% (0/5) placebo, p<0.01. The change from baseline in ability to tolerate tolerate 275mg peanut protein was 73% etokimab vs 0% placebo, p<0.01 (Chinthrajah 2019). |
| Change in median cumulative tolerated dose of peanut protein from baseline to day 15 | The impact of etokimab on ability to consume peanut is unclear because the certainty of evidence is very low. One small RCT found that etokimab increased the median dose of peanut tolerated at day 15 by 100mg vs 50mg increase for placebo (p not reported). | Very low | 1 RCT (n = 20) | One RCT found that adults receiving a single dose of intravenous etokimab (300mg/100 mL) were able to tolerate a higher median dose of peanut protein at 15 days compared to those receiving placebo:   - Etokimab: median cumulative dose tolerated on day 0 and 15: 175mg vs 275mg, p=0.001. - Placebo: median cumulative dose tolerated on day 0 and 15: 25mg vs 75mg, p=0.63.   Change in median peanut protein tolerated on day 15 compared to baseline: etokimab 100mg vs placebo 50mg, p not reported (Chinthrajah 2019). |
| Treatment emergent adverse events measured up to day 45 | The impact of etokimab on treatment emergent adverse events is unclear because the certainty of evidence is very low. One small RCT found that etokimab was well tolerated.  . | Very low | 1 RCT (n = 20) | One RCT found that a single dose of intravenous etokimab (300mg/100 mL) was well tolerated. 20% of the etokimab group (3/15) had mild adverse events compared with 40% of placebo (2/5). 60% of etokimab group had moderate events (9/15) versus 100% of placebo (5/5), p not reported. For etokimab, the most frequent adverse events was headache. For placebo, the most frequent treatment emergent adverse events were atopy-related (asthma, eczema, food allergy and allergic rhinitis) (Chinthrajah 2019). |
| Severe treatment emergent adverse events measured up to day 45 | The impact of etokimab on treatment emergent adverse events is unclear because the certainty of evidence is very low. | Very low | 1 RCT (n = 20) | One RCT of a single dose of intravenous etokimab (300mg/100 mL) found no severe or systemic adverse events in either the etokimab or placebo groups (p>0.05) (Chinthrajah 2019). |

**Evidence profile**

| Studies (participants) | Risk of bias | Inconsistency | Indirectness | Imprecision | Publication bias | Overall certainty |
| --- | --- | --- | --- | --- | --- | --- |
| **Ability to consume peanut at day 15** | | | | | | |
| 1 RCT (n = 20) | Reduced grade by one level due to moderate risk of bias | No grade change. One study only so not able to compare consistency of findings, but appears to be consistent internally across different measures of ability to consume peanut. | Reduced grade by one level. Study open to adults with wide range of symptoms, but severity of peanut allergy not specified so difficult to assess generalizability. People with severe reaction to screening were excluded. Median tolerated dose at baseline may not be representative. | Reduced grade by one level due to small sample size and very small number in control group (5). This results in wide confidence intervals for difference between groups. P-values not reported for differences in change from baseline between groups. | Uncertain. Single study available is sponsored by industry. Analyzed using pre-specified plan. | **Low**  Increased grade by one level due to large effect size. No reason to grade up based on dose response or potential effect of confounders. |
| **Change in median cumulative tolerated dose at day 15** | | | | | | |
| 1 RCT (n = 20) | Reduced grade by one level due to moderate risk of bias | No grade change. One study only so not able to compare consistency of findings, but appears to be consistent internally across different measures of ability to consume peanut. | Reduced grade by one level. Study open to adults with wide range of symptoms, but severity of peanut allergy not specified so difficult to assess generalizability. People with severe reaction to screening were excluded. Single country. | Reduced grade by one level due to small sample size and very small number in control group (5). This results in wide confidence intervals for difference between groups. P-values not reported for differences in change from baseline between groups. | Uncertain. Single study available is sponsored by industry. Analyzed using pre-specified plan. | **Very low**  No reason to grade up based on effect size, dose response or potential effect of confounders |
| **Treatment emergent adverse events up to day 45 and severe events** | | | | | | |
| 1 RCT (n = 20) | Reduced grade by one level due to moderate risk of bias | No grade change. Not able to compare consistency of findings. | Reduced grade by one level. Severity of peanut allergy not specified. People with severe reaction to screening were excluded which may impact the generalizability of data about adverse events. Only 9/20 people had measures as of day 45. Those who attended final clinic visit were self-selecting. Adverse events were self-reported, which can be variable. | Reduced grade by one level due to small sample size and very small number in control group (5). This results in wide confidence intervals for difference between groups and p-values not reported for outcomes. | Uncertain. Single study, sponsored by industry. Analysed according to pre-specified plan but p-values not reported for all safety outcomes, even though this was a core focus of the study. | **Very low**  No reason to grade up based on effect size, dose response or potential effect of confounders. |

1. Monotherapy with omalizumab

**Summary of findings**

| **Omalizumab** | | | | |
| --- | --- | --- | --- | --- |
| **People:** Children and adults with peanut allergy  **Settings:** Hospital, USA  **Intervention:** Omalizumab for 20-22 weeks every 2-4 weeks. Individualized dose based on weight and total IgE levels.  **Comparison:** Placebo | | | | |
| **Outcomes** | **Summary** | **Certainty of evidence** | **Number of studies (participants)** | **Findings of individual studies** |
| Ability to consume peanut: % able to consume 1g peanut protein at 24 weeks | The impact of omalizumab on people’s ability to consume 1g peanut is unclear because the certainty of evidence is very low. One RCT found no statistically significant difference between groups (p>0.05). | Very low | 1 RCT (n = 14) | One RCT found that the proportions who could tolerate 1g peanut protein were:   - 44% omalizumab (4/9) - 20% placebo (1/5)   This was not a statistically significant difference (p=0.324) (Sampson 2011). |
| Ability to consume peanut: change in threshold increase at 24 weeks compared to baseline | The impact of omalizumab on increases in thresholds is unclear because the certainty of evidence is very low. One RCT found no statistically significant difference between groups (p>0.05). | Very low | 1 RCT (n = 14) | One RCT found no statistically significant difference between groups in the change in maximum dose of peanut tolerated compared to baseline (p=0.054), despite positive trends (threshold increased average of 4.07 times for the placebo group compared to 80.9 times for omalizumab). There were no non-responders in the omalizumab group, but sample numbers were very small (5 placebo vs 9 omalizumab) (Sampson 2011). |
| Adverse events | The adverse event profile of omalizumab is unclear because the certainty of evidence is very low. One RCT found no statistically significant difference between omalizumab and placebo in mild, moderate or severe events (p>0.05).. | Very low | 1 RCT (n = 84) | One RCT found mild to moderate adverse events in 77% of the omalizumab group vs 89% of placebo (p>0.05). There were no systemic adverse events reported (Sampson 2011). |

**Evidence profile**

| Studies (participants) | Risk of bias | Inconsistency | Indirectness | Imprecision | Publication bias | Overall certainty |
| --- | --- | --- | --- | --- | --- | --- |
| **% able to consume 1g peanut protein** | | | | | | |
| 1 RCT (n = 14) | Reduced grade by one level due to moderate risk of bias, including early study termination. | No grade change. One study only so not able to compare consistency of findings. Appears to be internal consistency of measurement. | Reduced grade by one level as severity of allergy not clear. Single study in single country so questions about generalizability. | Reduced grade by one level due to lack of precision in estimates and no reporting of confidence intervals. Percentages based on very small number in each group. Numbers did not reach statistical power. Use per protocol analysis. | Reduced by grade by one level due to one published RCT only, even though other studies conducted. Study sponsored by industry and closed before completions. | **Very low**  No reason to grade up based on effect size, dose response or potential effect of confounders. |
| **Change in threshold increase from baseline** | | | | | | |
| 1 RCT (n = 14) | Reduced grade by one level due to moderate risk of bias, including early study termination. | No grade change. One study only so not able to compare consistency of findings. Appears to be internal consistency of measurement, but reporting approach not transparent | Reduced grade by one level as severity of allergy not clear. Single study in single country so questions about generalizability. | Reduced grade by one level due to lack of precision in estimates and no reporting of confidence intervals. Percentages based on very small number in each group. Numbers did not reach statistical power. Use per protocol analysis. | Reduced by grade by one level due to one published RCT only, even though other studies conducted. Study sponsored by industry and closed before completions. | **Very low**  No reason to grade up based on effect size, dose response or potential effect of confounders. |
| **Adverse events and systemic adverse events up to 20 weeks** | | | | | | |
| 1 RCT (n = 14) | Reduced grade by one level due to moderate risk of bias, including early study termination. Intended to randomize 150 patients but terminated at 14. Has an important effect on certainty given high proportion in both groups with adverse events. | No grade change. One study only so not able to compare consistency of findings Measurement approach uncertain. | Reduced grade by one level as severity of allergy not clear. Single study in single country. Adverse events were self-reported so subjective. | Reduced grade by one level due to lack of precision in estimates and reporting. P-values and confidence intervals not reported; appears no difference between groups. Percentages based on very small number in each group. | Reduced by grade by one level due to one published RCT only, even though other studies conducted. Study sponsored by industry and closed before completions. | **Very low**  No reason to grade up based on effect size, dose response or potential effect of confounders. |

1. Monotherapy with TNX-901

**Summary of findings**

| **TNX-901 four doses** | | | | |
| --- | --- | --- | --- | --- |
| **People:** Adults with moderate to severe peanut allergy  **Settings:** Hospital, USA  **Intervention:** 150mg, 300mg, or 450mg of TNX-901 subcutaneously every 4 weeks for 4 doses.  **Comparison:** Placebo | | | | |
| **Outcomes** | **Summary** | **Certainty of evidence** | **Number of studies (participants)** | **Findings of individual studies** |
| Ability to consume peanut: % able to consume 8g peanut flour | Subcutaneous TNX-901 every 4 weeks for 4 doses may not improve the ability to consume 8g peanut flour, but the evidence is of low certainty. One RCT found no statistically significant difference between any dose of TNX-901 and placebo (p>0.05). | Very low | 1 RCT (n = 84) | One RCT found that the proportion who could tolerate 8g peanut flour 2-4 weeks after treatment was:   - 0% 150mg TNX-901 - 21% 300mg TNX-901 - 24% 450mg TNX-901 - 4% placebo   Pairwise comparisons with placebo did not reach statistical significance at any threshold level (Leung 2003). |
| Ability to consume peanut: change in mean threshold dose compared to baseline | 450mg subcutaneous TNX-901 every 4 weeks for 4 doses may increase the ability to consume peanut, but the evidence is of low certainty. One RCT found a mean increase in threshold dose of 2627mg peanut flour compared to 710mg placebo (p<0.001). There was no statistically significant difference with lower doses of TNX-901. | Low | 1 RCT (n = 84) | One RCT compared 150mg vs 300mg vs 450mg subcutaneous TNX-901 vs placebo every 4 weeks for 4 doses. 2-4 weeks after the final dose, the mean increase in threshold compared to baseline was:   - 150mg TNX-901 913mg of peanut four - 300mg TNX-901 1650mg - 450mg TNX-901 2627mg - 710mg placebo   Only the difference between the 450mg dose and placebo was statistically significant (p<0.001). The test for trends in improvement with increasing doses was significant (p<0.001). The authors concluded that a 450mg dose of TNX-901 increased the threshold of sensitivity to peanut from about half a peanut at baseline (178mg) to almost nine peanuts by 18-20 weeks (2805mg) (Leung 2003). |
| Adverse events measured up to 20 weeks and systemic adverse events up to 20 weeks | The adverse event profile of TNX-901 is unclear because the certainty of evidence is very low. One RCT found that TNX-901 has a similar number and type of adverse events, including systemic adverse events, compared to placebo (p not reported). | Very low | 1 RCT (n = 84) | One RCT found that the incidence and type of adverse events was similar between 150mg, 300mg or 450mg subcutaneous TNX-901 vs placebo.  The total number of systemic adverse events reported (range 45 to 50 per group) and the number of patients who had systemic events (range 15 to 19 per group out of 19-21) were similar among the four groups. 13-14 out of 19-21 people per group had local injection site reactions (Leung 2003). |

**Evidence profile**

| Studies (participants) | Risk of bias | Inconsistency | Indirectness | Imprecision | Publication bias | Overall certainty |
| --- | --- | --- | --- | --- | --- | --- |
| **Mean increase in threshold dose** | | | | | | |
| 1 RCT (n = 84) | No grade change. Low risk of bias | No grade change. One study only so not able to compare consistency of findings, but appears to be consistent internally across different measures of ability to consume peanut. | Reduced grade by one level due to difference between groups in baseline tolerance thresholds which may affect generalizability. Applicable to those with moderate to severe peanut allergy, and only in single country. Single study only. | Reduced grade by two levels due to limited data. Data were reported as graphs rather than exact numerical measures, so difficult to confirm precision. P-values are reported for differences. The higher the dose, the larger the width of confidence intervals. Numbers in each dose group relatively small (19-21). Half of groups did not reach level required for power calculation. | No grade change. Bias possible as one published study only. Study sponsored by industry, but independent analysis and decision-making. | **Very low**  No reason to grade up based on effect size or potential effect of confounders. There is a dose response relationship but one study only. |
| **% able to consume 8g peanut flour** | | | | | | |
| 1 RCT (n = 84) | No grade change. Low risk of bias | No grade change. One study only so not able to compare consistency of findings. Appears to be consistent internally across different measures of ability to consume peanut, but this outcome was not statistically significant. | Reduced grade by one level due to difference between groups in baseline tolerance thresholds which may affect generalizability. Applicable to those with moderate to severe peanut allergy, and only in single country. Single study only. | Reduced grade by two levels due to limited data. Data were reported as graphs rather than exact numerical measures. P-values are reported for differences but not confidence intervals. Numbers in each dose group relatively small (19-21). | No grade change. Bias possible as one published study only. Study sponsored by industry, but independent analysis and decision-making. | **Low**  Graded up one level based on dose response relationship trends.  No reason to grade up based on effect size or potential effect of confounders. |
| **Adverse events and systemic adverse events up to 20 weeks** | | | | | | |
| 1 RCT (n = 84) | No grade change. Low risk of bias | No grade change. One study only so not able to compare consistency of findings. Outcome measured every 4 weeks and appears internally consistent. | Reduced grade by one level as applicable to those with moderate to severe peanut allergy, and only in single country. Single study only. Some adverse events were self-reported so subjective. | Reduced grade by two levels due to limited data reporting. P-values and confidence intervals not reported. Numbers in each dose group relatively small (19-21). Uncertain the total number of people who had any events overall or proportion of doses overall. | No grade change. Bias possible as one published study only. Study sponsored by industry, but independent analysis and decision-making. | **Very low**  No reason to grade up based on effect size, dose response or potential effect of confounders. |
